# Injection Site Erythema in a Patient on Therapeutic Anticoagulation with Low Molecular Weight Heparin after Mechanical Aortic Valve Replacement: A Rare Presentation of Heparin- and Protamine-Induced Thrombocytopenia

**DOI:** 10.1155/2020/4503598

**Published:** 2020-04-10

**Authors:** Caroline Holaubek, Paul Simon, Sabine Eichinger-Hasenauer, Franz Gremmel, Barbara Steinlechner

**Affiliations:** ^1^Division of Cardiothoracic and Vascular Anesthesia and Intensive Care, Medical University of Vienna, Vienna, Austria; ^2^Division of Cardiac Surgery, Medical University of Vienna, Vienna, Austria; ^3^Division of Hematology and Hemostaseology, Department of Medicine I, Medical University of Vienna, Vienna, Austria

## Abstract

Previous exposition to heparin and protamine in patients undergoing cardiopulmonary bypass and postoperative therapeutic anticoagulation with LMWH may lead to the development of heparin-induced thrombocytopenia (HIT) and/or protamine-induced thrombocytopenia (PIT). This case deals with a rare clinical presentation of circulating IgG antibodies against heparin/platelet factor 4 complexes and heparin/protamine complexes after cardiac surgery. Ensuing purpura and skin necrosis (blisters) at the injection sites of LMWH and clinical symptoms improved rapidly after replacement of LMWH by an alternative anticoagulant. The aim of this report is to draw attention to the several different clinical manifestations of heparin- and/or protamine-induced thrombocytopenia and shows a possible course of treatment and recovery.

## 1. Introduction

Therapeutic low molecular weight heparin (LMWH) after mechanical aortic valve replacement and previous exposition to heparin and protamine during cardiac surgery on cardiopulmonary bypass (CPB) may trigger heparin-induced thrombocytopenia (HIT) and/or protamine-induced thrombocytopenia (PIT). HIT is caused by the formation of IgG antibodies against immunogenic heparin/platelet factor 4 complexes, whereas PIT is caused by antibodies against protamine alone and/or protamine/heparin complexes [1]. The diagnosis of HIT is based on clinical signs and symptoms including a decline of platelets and thromboembolic complications supported by confirmatory laboratory testing [2]. The diagnosis of PIT is still a rarity although an increase of seropositive patients after cardiac surgery has been reported. An associated higher risk of early thrombosis and thrombocytopenia was suspected [1].

## 2. Case Report

**Figure 1:**
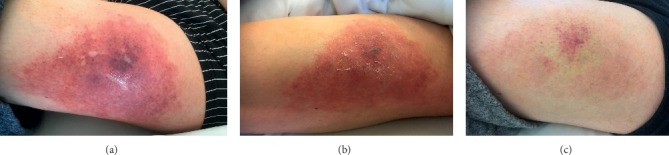
Clinical illustrations. (a) 7th postoperative day. (b) 15 hours after switch. (c) 11th postoperative day.

A 54-year-old male patient with severe aortic valve stenosis (strongly calcified bicuspid valve) undergoing mechanical aortic valve replacement developed injection-site erythemas after 5  days of therapeutic LMWH (enoxaparin 40  mg BID). An allergic hypersensitivity reaction to heparin was suspected, and enoxaparin was switched to another LMWH (fraxiparin) followed by intravenous unfractionated heparin since hypersensitivity reactions continued. In parallel, oral anticoagulation with a vitamin K antagonist (phenprocoumon) was started at a dose of 9 mg on 2 consecutive days. On the seventh day after surgery, all LMWH injection sites became increasingly painful and inflamed and showed necrotic central lesions and blisters, with the first site at the left thigh being the most severely affected (Figure 1). In addition, the patient described a tingling sensation in the tips of his fingers and toes.

**Figure 2:**
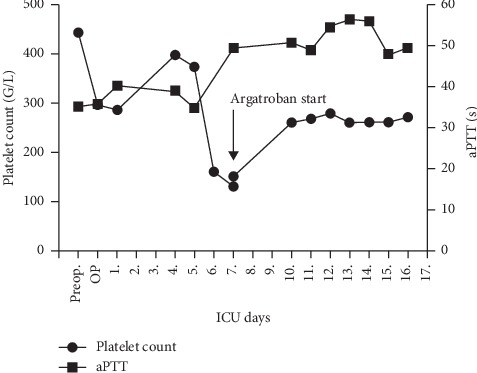
Course of platelet count (150–350 G/L) and aPTT values (27–41 sec).

There was a perioperative drop in the platelet count with recovery 4 days after surgery (398 G/l). On day 6, platelet count decreased to 160 G/l but remained within the normal range (Figure 2). However, based on the criteria of the 4 Ts score, more than 50% drop of platelet count together with the onset of the symptoms, platelet count fall between 5 and 10 days after the start of heparin, gangrenous skin lesion, and no apparent other causes for thrombocytopenia indicate a high probability for HIT (8 points).

**Figure 3:**
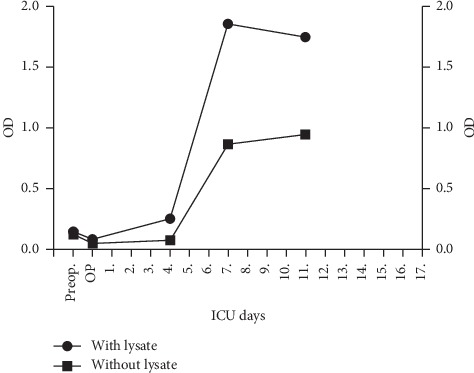
Antibody levels in our patient.

Heparin and phenprocoumon were immediately stopped, vitamin K was administered at an INR of 1.5, and argatroban at an initial dosage of 0.15 *μ*g/kg/min IV was started as an alternative anticoagulant. The occurrence of HIT correlates with higher optical density in specific ELISA tests [3]. On the seventh postoperative day, ELISA tests showed highly positive antibody levels/OD, 2.0 with lysate (HIT), and 0.8 without lysate (PIT) (optical densities from Zymutest® HIA IgG enzyme-linked immunosorbent assay (ELISA) test (Hyphen Biomed)) [4] (Figure 3). Platelet count increased steadily thereafter, and the local injection sites recovered (Figure 1). After normalization of the platelet count, phenprocoumon was restarted, and argatroban was discontinued once an INR above 2.0 was reached.

## 3. Discussion

We present a case of HIT with several unusual but very distinct features. Patient developed HIT while receiving LMWH. Compared to UFH, prevalence of HIT during LMWH is very rare [5]. However, the risk of HIT increases during certain high-risk situations including cardiac surgery. Whether switching to UFH aggravated the clinical syndrome remains to be discussed. Skin reactions to heparin are mostly allergic hypersensitivity reactions. An association with HIT has been reported [5].

Low platelet count is the hallmark in establishing the diagnosis of HIT. Many patients present with increased platelet counts particularly after surgery. It is important to note that not only a platelet count numerically below the lower limit of the normal range but also a decline of more than 50% in platelet number may also be an indicator of HIT.

In patients with confirmed HIT, oral anticoagulation with a vitamin K antagonist is contraindicated as this may cause gangrenous lesions usually in the fingers or toes. Interestingly, in our patient, gangrenous lesions started in the erythematous injection sites. It can be surmised that the tingling sensations in the tips of fingers and toes were related to impaired circulation in the microvasculature.

Protamine is routinely used after cardiac surgery to reverse the anticoagulant effects of heparin. Platelet-activating anti-protamine-heparin antibodies show several similarities with anti-platelet factor 4-heparin antibodies and are a potential risk factor for early postoperative thrombosis [1].

Upon suspicion of HIT and later confirmed by antibody levels with and without lysate (Figure 3), all heparin was stopped. We decided for the parenteral thrombin inhibitor argatroban as an alternative anticoagulant because of its availability and our vast clinical experience with this thrombin inhibitor in particular.

Clinical symptoms and platelet count improved/normalized with the alternative anticoagulant.
